# A case of testicular cancer in a long‐term hydranencephaly survivor with undescended testes

**DOI:** 10.1002/iju5.12720

**Published:** 2024-04-01

**Authors:** Wataru Hirata, Toshiaki Shinojima, Kotaro Yokota, Rei Kin, Taketo Yamada, Hirotaka Asakura

**Affiliations:** ^1^ Department of Urology Saitama Medical University Moroyama Saitama Japan; ^2^ Department of Pathology Saitama Medical University Moroyama Saitama Japan

**Keywords:** cerebral palsy, hydranencephaly, testicular cancer, undescended testes

## Abstract

**Introduction:**

The development of malignant tumors in patients with hydranencephaly is extremely rare. We describe the first case of testicular cancer that developed in the undescended testes of a long‐term survivor of hydranencephaly.

**Case presentation:**

A 32‐year‐old man with severe cerebral palsy due to hydranencephaly was referred to our department for the evaluation of a subcutaneous lump in the lower right abdomen. He was a long‐term survivor of hydranencephaly. After confirming the diagnosis of right testicular cancer originating in his undescended testes, surgical resection was performed. Pathological examination revealed a mixed‐type germ cell tumor.

**Conclusion:**

The decision‐making process for treating malignant tumors, like testicular cancer, in adults with severe cerebral palsy can be challenging. Clinical ethics consultation could be helpful in avoiding treatment delays.

**Abbreviations & Acronyms**
AFP: α‐fetoprotein
CP: cerebral palsy
CT: computed tomography
H&E: hematoxylin and eosin
hCG: human chorionic gonadotropin
LDH: lactate dehydrogenase
UDT: undescended testes

**Keynote message**

Patients with cerebral palsy due to hydranencephaly very rarely survive for a long time. Cerebral palsy is a risk factor for undescended testes. Our case highlights the possibility of testicular cancer development even in severe cerebral palsy patients with undescended testes. The clinical decision‐making process of such patients is challenging.

## Introduction

Hydranencephaly is a rare condition characterized by an almost complete absence of the cerebral hemispheres since birth.^1^ Many patients with hydranencephaly die within the first year of life, and cases with long‐term survival beyond the age of 20 years are very rare. Among the various comorbidities of hydranencephaly, CP is a risk factor for UDT.^2^ In the present case report, we describe a case of testicular cancer in a hydranencephaly patient with UDT who survived long‐term.

## Case presentation

**Fig. 1:**
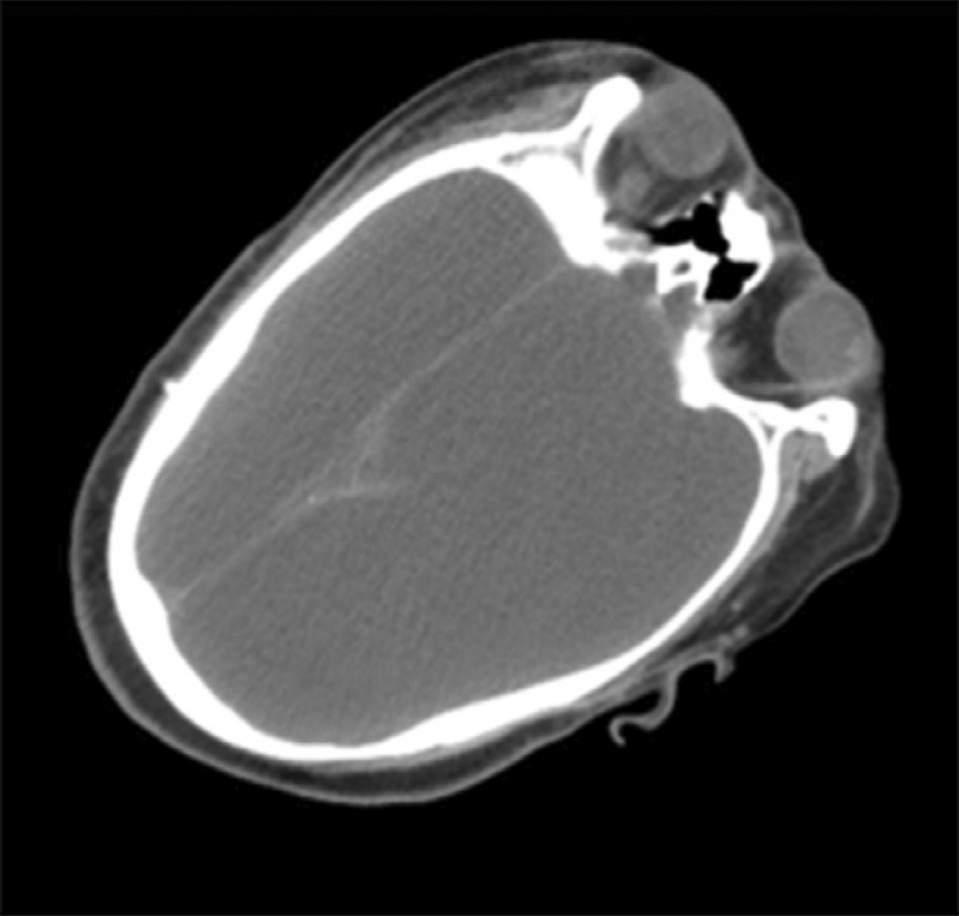
Brain CT scan showing complete replacement of the cerebral hemispheres with fluid.

**Fig. 2:**
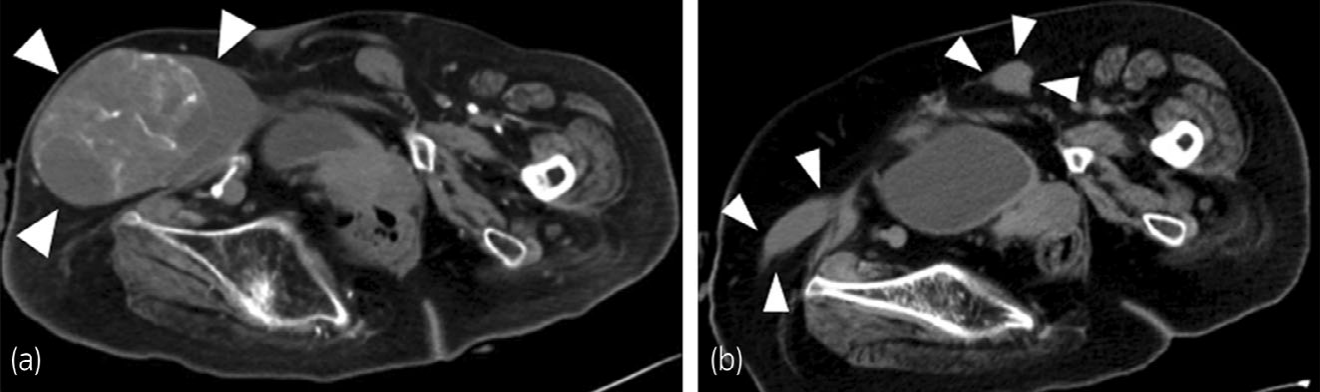
Pelvic CT images. (a) A 10‐cm well‐circumscribed oval tumor (white arrowheads). (b) An image of the bilateral UDT (white arrowheads) obtained 4 years ago.

A 32‐year‐old man with severe developmental delay and spastic quadriplegia due to hydranencephaly (Fig. 1), who had been cared for by a facility for persons with severe motor and intellectual disabilities, was referred to our dermatology department for the evaluation of a subcutaneous lump in the lower right abdomen. Abdominal CT revealed a well‐defined 10 × 5.5‐cm solid tumor with a mix of solid and cystic components (Fig. 2a). Given that a CT scan taken 4 years previously demonstrated bilateral UDT (Fig. 2b), the origin of the tumor was suspected to be the right testis located within the lateral area of the superficial inguinal pouch. He was immediately referred to our urology department for further evaluation. At the initial visit, blood tests revealed elevated tumor markers with AFP at 10 481 ng/mL, hCG at 24.6 IU/L, and LDH at 232 U/L, leading to the diagnosis of right testicular cancer. The tumor on palpation was not fixed to the abdominal wall, and the imaging studies showed no metastasis.

Owing to his high risk for complications associated with general anesthesia and poor performance status, which made him unfit for any adjuvant treatment, the initial treatment plan proposed by urologists was observation and best supportive care. During follow‐up, the tumor visibly increased in size, and his caregivers were hesitant to leave it untreated. After repeated discussions with his mother and attending physician in the facility, we eventually planned to perform a surgical resection of the tumor; however, by then, it had already been 4 months since the initial consultation. A preoperative CT scan demonstrated that the tumor diameter increased to 11 × 7 cm, and the AFP and LDH levels were elevated to 17 668 ng/mL and 335 U/L, respectively, but there was still no evident metastatic or nodal disease.

**Fig. 3:**
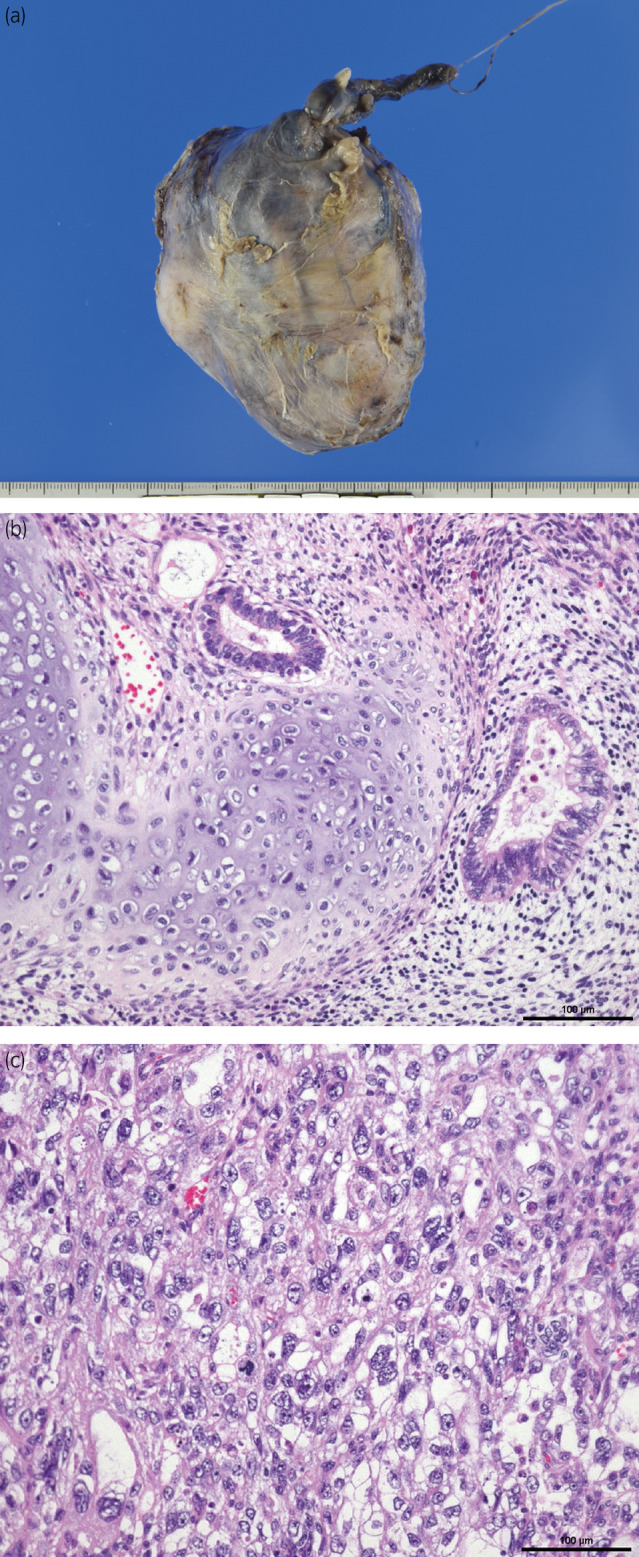
Gross and microscopic findings of testicular tumor. (a) A complete resection of the tumor with the testis and spermatic cord. (b) A magnification view of the immature teratoma component shows the presence of tubular structures and cartilage (H&E staining, ×200; scale bar = 100 μm). (c) A solid pattern of embryonal cell carcinoma (H&E, ×200; scale bar = 100 μm).

A right lower abdominal oblique incision along the tumor's long axis was made from the junction of the testicular tumor and spermatic cord, whose location was confirmed by ultrasonography. The spermatic cord was first lifted and clamped near the testis. The tumor was then mobilized from the abdominal wall, allowing the exposure of the anterior wall of the inguinal canals in the operative field. Subsequently, high ligation of the spermatic code was performed as usual (Fig. 3a).

Pathological examination revealed a mixed‐type germ cell tumor comprising an immature teratoma (Fig. 3b) and an embryonal carcinoma (Fig. 3c), with negative microscopic margins and negative lymphovascular invasion. He was discharged on the 23rd postoperative day. At the 5‐month follow‐up, no obvious metastatic lesions were observed on imaging, and the AFP, hCG, and LDH levels decreased and were within the normal range.

## Discussion

Hydranencephaly is caused by impaired blood flow in the internal carotid artery during fetal development, leading to failure in the development of the cerebral hemispheres. It is accompanied by severe motor disabilities due to CP and symptoms from increased intracranial pressure.^1^ Most newborns die within a few weeks or months; however, some exceptional cases survive beyond 20 years of age. A literature review of 37 articles describing 76 patients with hydranencephaly reported only two adult patients (22 and 32 years) between 2000 and 2012, and our case might be one of the most long‐term survivors of hydranencephaly.^3^, ^4^ Moreover, to the best of our knowledge, this is the first report of testicular cancer in a male with hydranencephaly.

CP is a risk factor for UDT with a prevalence of approximately 24%–54%,^2^ which is more than 10 times higher than the usual incidence. A previous report highlighted the role of muscle spasticity in the etiology of UDT in boys with CP. The shortening of the cremaster muscle has been theorized to account for postnatal testicular ascent.^5^ A recent study on several CP patients from a single center also supported this theory by demonstrating a strong association between UDT and spasticity.^2^ In the present case, an additional factor other than muscle spasticity may have been involved in his UDT, because they were shifted far laterally within the superficial inguinal pouch.

UDT is a significant risk factor for testicular cancer with a relative risk of 2.75 to 8 among patients with UDT,^6^ and it seems to occur most frequently in the 20s, followed by the 30s and 40s.^7^ One of the important rationales for UDT treatment is to prevent testicular cancer from developing in adulthood. These rationales should not be different between boys with CP and those without neurological impairment. However, the decision‐making process in cases of UDT with CP is sometimes difficult, not only because the risk of anesthesia and life expectancy vary among patients, but also because such cases usually involve challenging ethical issues.^8^ To practice in the best interest of the patient, clinicians, and family members should consider all options and weigh the potential benefit of treatment (orchiopexy or orchiectomy) against its risks. Sometimes, a disagreement might exist between the CP family, care provider, and medical specialist.^8^

In the present case, the prognosis of hydranencephaly was generally so poor that it seemed obvious that UDT was not a prioritized physical problem. However, once the patient developed testicular cancer, we could not find any reviews or case reports providing the optimal management of testicular cancer in severe CP patients. His condition was not manageable, and it took some time to reach a consensus on the treatment among his attending physician, family, and specialists. Recently, there has been an increasing acknowledgment of the importance of focusing on the ethical dimension of clinical decision‐making.^9^ Clinical ethics consultation is an elegant tool for wise and open decision‐making in ethically difficult cases,^8^ and a clinical ethics committee at our institution should have been asked to share our case.

## Conclusion

The decision‐making process for treating malignant tumors, like testicular cancer, in adults with severe CP can be challenging. Clinical ethics consultation could be helpful in avoiding treatment delays.

## Author contributions

Wataru Hirata: Writing – original draft. Toshiaki Shinojima: Writing – review and editing. Kotaro Yokota: Writing – review and editing. Rei Kin: Writing – review and editing. Taketo Yamada: Supervision. Hirotaka Asakura: Supervision.

## Conflict of interest

The authors declare no conflict of interest.

## Approval of the research protocol by Institutional Reviewer Board

Not applicable.

## Informed consent

Written informed consent was obtained.

## Registry and the Registration No. of the study/trial

Not applicable.

## References

[1] Omar AT III , Manalo MKA , Zuniega RRA , Reyes JCB , Brillante EMB , Khu KJO . Hydranencephaly: clinical features and survivorship in a retrospective cohort. World Neurosurg. 2020; 144: e589–e596. 32916366 10.1016/j.wneu.2020.09.029

[2] Barthold JS , Wintner A , Hagerty JA , Rogers KJ , Hossain MJ . Cryptorchidism in boys with cerebral palsy is associated with the severity of disease and with co‐occurrence of other congenital anomalies. Front. Endocrinol. (Lausanne) 2018; 9: 151. 29713311 10.3389/fendo.2018.00151 PMC5911456

[3] Cecchetto G , Milanese L , Giordano R , Viero A , Suma V , Manara R . Looking at the missing brain: hydranencephaly case series and literature review. Pediatr. Neurol. 2013; 48: 152–158. 23337012 10.1016/j.pediatrneurol.2012.10.009

[4] Sen K , Kaur S , Stockton DW , Nyhuis M , Roberson J . Biallelic variants in LAMB1 causing hydranencephaly: a severe phenotype of a rare malformative encephalopathy. AJP Rep. 2021; 11: e26–e28. 33542858 10.1055/s-0040-1722728 PMC7850915

[5] Smith JA , Hutson JM , Beasley SW , Reddihough DS . The relationship between cerebral palsy and cryptorchidism. J. Pediatr. Surg. 1989; 24: 1303–1305. 2574233 10.1016/s0022-3468(89)80572-x

[6] Wood HM , Elder JS . Cryptorchidism and testicular cancer: separating fact from fiction. J. Urol. 2009; 181: 452–461. 19084853 10.1016/j.juro.2008.10.074

[7] Kikuchi Y , Irisawa S , Suzuki H , Ishii N , Numasawa K , Imamura A . Undescended testis cancer: report of two cases—review of the literature of 179 cases in Japan. Hinyokika Kiyo 1989; 35: 1791–1793. 2575353

[8] Springer A . Optimal management of undescended testis in boys with cerebral palsy. A debate. Sex. Dev. 2019; 13: 20–25. 30703771 10.1159/000496463

[9] Ignatowicz A , Slowther AM , Bassford C , Griffiths F , Johnson S , Rees K . Evaluating interventions to improve ethical decision making in clinical practice: a review of the literature and reflections on the challenges posed. J. Med. Ethics 2023; 49: 136–142. 35241628 10.1136/medethics-2021-107966

